# TRP channel-related LncRNAs, AC092535.4 and LINC01637, as novel prognostic biomarkers for uveal melanoma

**DOI:** 10.3389/fgene.2024.1441732

**Published:** 2024-07-23

**Authors:** Min Zhang, Jinglan Ni, Dongyue Liu, Yubo Cui, Xiaochen Ma, Jun Zhao

**Affiliations:** ^1^ The Second Clinical Medical College, Jinan University, Shenzhen, Guangdong, China; ^2^ Department of Ophthalmology, Shenzhen People’s Hospital, The Second Clinical Medical College of Jinan University and The First Affiliated Hospital of Southern University of Science and Technology, Shenzhen, Guangdong, China

**Keywords:** transient receptor potential channel, uveal melanoma, lncRNA, immune microenvironment, bioinformatics, prognosis

## Abstract

**Introduction:**

Transient receptor potential (TRP) channels function as cellular sensors with a broad impact, and their dysregulation is linked to numerous cancers. The influence of TRP channel-related long noncoding RNAs (TCRLs) on uveal melanoma (UM) remains poorly understood.

**Methods:**

We employed bioinformatics to examine the RNA-seq data and relevant clinical information of UM in the TCGA databases. By implementing coexpression analysis, we identified differentially expressed TCRLs. Using univariate Cox regression analysis, selection operator (LASSO) algorithm and stepwise regression, five key prognostic biomarkers were chosen. The high- and low-risk groups were divided based on the risk scores. Afterwards, the prediction performance of the signature was evaluated by receiver operating characteristic (ROC) curve and Kaplan-Meier (K-M) survival analysis. The functional enrichment analysis of TCRLs was also investigated. Following that, we examined immune cell infiltration, immune checkpoint expression, and tumor immune microenvironment between patients in high and low risk groups. TCRLs were validated using Random forests and multifactor Cox analysis. Candidate biomarkers were identified and screened. Finally, the effects of the candidate biomarkers on the proliferation, migration and invasion of UM cells were detected by CCK-8 assay, migration assay and perforation invasion assay.

**Results:**

The risk score generated by five TCRLs demonstrated robust predictive power. The high-risk group exhibited a poorer prognosis, increased immune cell infiltration, and an active tumor immune microenvironment compared to the low-risk group. Furthermore, two TCRLs of risk score, AC092535.4 and LINC01637, were screened to multiplex modelling. The *in vitro* experiments demonstrated that UM cells were suppressed following AC092535.4 or LINC01637 knockdown.

**Discussion:**

Two TCRLs, AC092535.4 and LINC01637, serve as novel prognostic biomarkers for uveal melanoma and may present potential therapeutic targets.

## 1 Introduction

Uveal melanoma (UM) is the most common primary intraocular malignancy. Approximately 30% of UM patients do not present any clinical symptoms that can be detected during routine eye examinations (Damato and Damato, 2012). The rate of distant metastasis is as high as 50% (Kujala et al., 2003). Metastatic UM patients receiving aggressive treatments have a median survival of only 10–13 months (Khoja et al., 2019). Currently, the treatment of UM is mainly surgical resection supplemented with radiotherapy (Branisteanu et al., 2021). However, current treatment outcomes are unsatisfactory, and the long-term prognosis is still dismal (Toro et al., 2021). The limitations of the treatment of UM motivated us to search for novel prognostic markers.

Transient receptor potential (TRP) channels—a superfamily of nonselective cation channels—including TRPM, TRPV, and four others (Wu et al., 2010; Koivisto et al., 2021). TRP channels are extensively distributed in mammals, playing crucial roles in various cellular functions as well as the development of diseases (Yuan et al., 2003; Santoni and Farfariello, 2011). Initially, altering the expression of members of the TRPM and TRPV families can change the membrane-associated calcium-ion-dependent proliferative response, triggering intracellular cascades (Tsavaler et al., 2001; Nabissi et al., 2010). Additionally, TRP channels exhibit upregulation in various types of cancer, including melanoma as well as cancers of the prostate, breast, thyroid, colon, and ovaries. This is due to the fact that it promotes cancer progression not only by resisting apoptotic cell death but also by promoting cancerous cellular proliferation and differentiation (Kiselyov et al., 2007; Nilius et al., 2007; Santoni et al., 2011; Rivas et al., 2020). It is worth noting that the TRP channels expressed in melanoma is particularly abundant. TRPM5 is overexpressed in melanoma (Loo et al., 2017). TRPV2 is overexpressed in melanoma cell lines with N-RAS mutations (Bloethner et al., 2005). The patient with melanoma-associated retinopathy diagnostics positive for TRPM1 antibody (Shinohara et al., 2021). This indicates that TRP channels may possess oncogenic properties and could potentially aid in the detection of melanoma. Furthermore, an increasing amount of evidence indicates that TRP channels impact the progression of UM. TRPM8 inhibits the progression of uveal melanoma by blocking the transactivation of TRPV1 by VEGF (Walcher et al., 2018). The expression of TRPM4 and TRPV2 has been confirmed to be increased in UM (Wang et al., 2022). This indicates that TRP channels are a promising therapeutic target for uveal melanoma. Interestingly, there have been no reports on TRP channel-related lncRNAs (TCRLs) in UM to date.

LncRNAs are non-protein-coding transcripts with a length of more than 200 nucleotides (Lian et al., 2016). The regulation of gene expression is aided by lncRNAs, such as chromatin modification (Schmitz et al., 2016), gene transcription (Melé and Rinn, 2016), and posttranslational modification (Lin et al., 2016). Hence, lncRNAs participate in cellular processes, such as the cell cycle and cell differentiation (Kitagawa et al., 2013; Delás et al., 2017). It is not difficult to understand that lncRNAs are implicated in the progression of cancer. LncRNAs contain oncogenes and tumor suppressor genes which enforce expression or depletion can induce alterations in the tumor phenotype (Wang et al., 2013; Yarmishyn and Kurochkin, 2015). Therefore, lncRNAs can serve as biomarkers for the diagnosis and prognosis of many cancers. However, 98% of the targets for antitumor treatments are located in the non-coding region, indicating the unknown mechanism of action of lncRNAs in tumors (Guzel et al., 2020).

In this study, we aim to systematically identify novel TCRLs related to UM, which provide a fresh perspective for new treatment strategies for UM.

## 2 Materials and methods

### 2.1 Data acquisition

High-throughput RNA sequencing and clinical data from 80 UM patients were downloaded from the Cancer Genome Atlas (TCGA) database (https://portal.gdc.cancer.gov/). Gene expression profiles (FPKM values) were normalized. Patients were excluded if their survival duration was less than 30 days or if their pathological stage was unknown to eliminate potential bias. For further analysis, 74 samples were considered, which were then randomly divided into training and testing sets of the same sample size using the R package “caret.” The training set was used to build the model, while the testing set and the entire sample were used to validate the model. Considering that the TCGA database is open access, approval by an ethics committee was not required.

### 2.2 Identification of TCRLs

The REACTOME_TRP_CHANNELS pathway in the MsigDB database (https://www.gsea-msigdb.org/gsea/msigdb) and the inflammatory mediator regulation of the TRP channel pathway in the KEGG database (https://www.genome.jp/kegg/) supplied data for 120 TRP channel-related genes (TCRGs). The GENECODE database (https://www.gencodegenes.org/human/) provided annotation files for lncRNAs and protein-coding genes. The R package “limma” was used, and screening criteria to identify TCRLs were set as a Pearson correlation coefficient value greater than 0.4 and *p* < 0.001. The co-expression network to demonstrate the interrelations among mRNAs and lncRNAs was constructed using the R package “igraph.”

### 2.3 Construction of TCRL signature

The univariate Cox regression and LASSO Cox regression were performed on the training set to select TCRLs for building a risk-scoring model. Plots were drawn using the R package “ggplot2.” The risk scores for all clinical cases were calculated using the following formula: Risk score = ∑Exp _TCRLs_ × Coef _TCRLs_. Exp is the associated expression value, and Coef is the coefficient of the multiple Cox regression analysis of TCRLs. The median risk score was regarded as the cutoff point to divide the training set, the testing sets, and the entire sample into two subgroups (high-risk and low-risk groups). A Sankey diagram was constructed to illustrate the relationship between TCRLs of the risk score and their related TCRGs using the R package “ggalluvial.”

### 2.4 Validation of TCRL signature

Univariate and multivariate independent prognostic analyses were conducted to determine whether the signature could be used as an independent factor to predict the prognosis of patients using the R package “survival.” The predictive accuracy of TCRL signature was assessed using risk curve analysis, receiver operating characteristic (ROC) curve analysis, Kaplan-Meier (K-M) survival analysis, and PFS analysis using the R packages “pheatmap,” “timeROC,” “survival,” and “survminer,” respectively. Based on clinicopathological characteristics, including age, gender, stage, and T, a K-M survival analysis was used to assess the prognostic value.

### 2.5 Development of a prognostic nomogram

By combining the risk score with clinical traits, a prognostic nomogram for patients with UM was developed to assess 1-, 2-, and 3-year survival rates using the R package “rms.” The accuracy of the prognostic nomogram was validated by calibration curves.

### 2.6 Enrichment analysis

Differentially expressed genes (DEGs) between two risk groups were analyzed using the R package “limma” following the screening criteria of *p* < 0.05. GO and KEGG enrichment analyses of DEGs were carried out to explore their biological functions. The results were visualized using circos plot and bubble diagrams using the R packages “circlize” and “ggplot2,” respectively.

### 2.7 Unsupervised cluster analysis

Unsupervised cluster analysis was performed based on the k-means clustering algorithm using the R package “ConensusClusterPlus” to determine TCRL-related subgroups. We performed PCA and t-SNE analysis using the R package “Rtsne.” K-M survival curves of two clusters were drawn to find the overall survival differences between clusters.

### 2.8 Immune microenvironment analysis

To verify the relationship between risk scores and immune components, a bubble plot was drawn based on different algorithms using the R package “ggplot2.” The difference in immune cell components based on different algorithms and the difference in immune-related functions were depicted by boxplots and heatmaps using the R package “ggplot2” and “ggpubr” in the case of between-risk groups and using the R package “heatmaps” in the case of between clusters, respectively. We further investigated the differential expression of immune checkpoints by risk group and cluster. Immune, stromal, and ESTIMATE scores of UM patients were calculated using ESTIMATE algorithm.

### 2.9 Immunotherapy and drug sensitivity analysis

The tumor immune dysfunction and exclusion (TIDE) score files of the UM patients were obtained from the TIDE website (http://tide.dfci.harvard.edu). Violin plots were generated using the “vioplot” package to demonstrate the differences in TIDE between risk groups. The R package “pRRophetic” was used to evaluate the sensitivity (IC50) in risk groups, which was used to determine how sensitive UM patients were to medication treatment. The Wilcoxon signed-rank test was used to compare the IC50 values between the groups.

### 2.10 Validation of multiple modelling methods

The package “randomForest” was employed to build a Random Forest model to filter genes. Genes with the utmost significance were screened for the subsequent analysis. Multivariate Cox analysis was used to identify differentially expressed genes associated with survival outcomes, controlling for relevant covariates. Genes with significant associations were selected for further analysis.

### 2.11 Confirmatory cell experiments

#### 2.11.1 Cell culture and siRNA transfection

Human invasive UM cell line (MUM2B, iCell, China) was cultivated in RPMI 1640 mediums. Small interfering and negative control RNA (si-NC, Ribobio, China) were used in knockdown experiments. The MUM2B cells were harvested 48 h after transfection for RNA extraction and processed for functional assays. The siRNA sequences are listed in Supplementary Table S1.

#### 2.11.2 RNA collection and quantitative real-time PCR

Total RNA was extracted from different transfected MUM2B cells using TRIzol (Invitrogen, CA, United States). Quantitative real-time PCR (qRT-PCR) was used to verify transfection efficiency. A list of the primers used can be found in Supplementary Table S2. The qRT-PCR reaction was prepared using the riboSCRIPTTM mRNA/lncRNA qRT-PCR Starter Kit (Ribobio, China), following the manufacturer’s instructions.

#### 2.11.3 Cell proliferation

MUM2B cells were seeded at a density of 2000 cells per well in 96-well plates. Cell Counting Kit-8 (CCK8, Biosharp, China) assays were performed 24, 48, and 72 h after transfection to determine cell proliferation. 10 μL CCK8 was added to the medium of each well during measurement, and the absorbance was measured at 450 nm after incubation for 45 min.

#### 2.11.4 Cell invasion assays

A total of 4 × 10^4^ cells were added to Matrigel-coated upper transwell chambers for the invasion assay. After 48 h, cells were immobilized by 4% paraformaldehyde for 15 min and stained with 0.1% crystal violet for 5 min. The number of stained MUM2B cells was counted in three randomly chosen fields.

#### 2.11.5 Scratch test

In scratch test assay, MUM2B cells were cultured in 6-well plates. Transfections were performed when cells reached 40% confluence. The scratch was made by a 200 μL pipette tip when the cell confluence grew to 80%. The wound was observed after 24 h, and the scratch area was measured using ImageJ software.

### 2.12 Statistical analysis

T-test was applied to examine age differences. A chi-square test was performed to analyze the differences in other clinical characteristics. The statistical analysis was performed using the R software, version 4.2.1. The *p*-value for statistical significance was set at 0.05 unless otherwise stated.

## 3 Results

**FIGURE 1:**
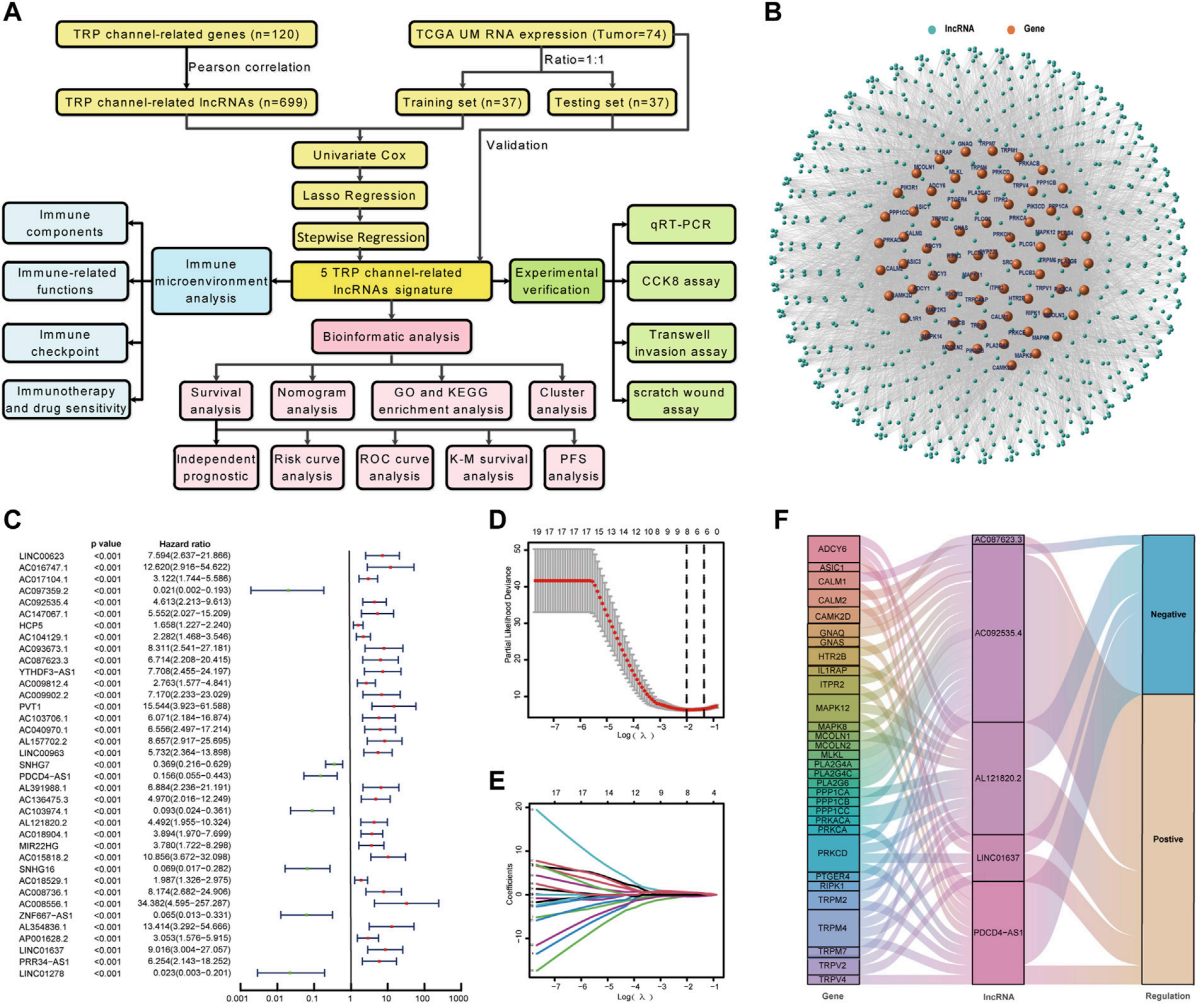
Construction of TCRL signature. **(A)** Flow diagram of the present study. **(B)** A co-expression network was constructed between TCRLs and their related mRNAs. **(C)** A forest map of TCRLs using univariate Cox analysis (*p* < 0.001). **(D)** LASSO coefficients produced by LASSO regression analysis. **(E)** LASSO coefficient profiles of eight TCRLs. **(F)** The Sankey diagram illustrates the connection between five TCRLs and their related TCRGs.

The construction and validation of the TCRL signature followed the flowchart represented in Figure 1A. Based on 120 TCRGs (Supplementary Table S3), we identified 699 TCRLs in TCGA-UM data with a Pearson correlation coefficient value greater than 0.4 and *p* < 0.001. A co-expression network was constructed between TCRLs and their related mRNA (Figure 1B). A total of 74 patients were divided into training and testing sets of 37 patients each based on a random process. Randomization created groups that did not differ on demographic and clinicopathological characteristics (Supplementary Table S4). In the training set, 37 TCRLs with prognostic values were filtrated through univariate Cox regression analysis (Figure 1C). Subsequently, LASSO Cox analysis was performed to avoid model overfitting, and the results indicated that eight TCRLs were identified from the original 37 TCRLs (Figures 1D, E). Finally, stepwise regression identified five TCRLs (AC092535.4, AC087623.3, PDCD4-AS1, AL121820.2, and LINC01637) as prognostic biomarkers based on the AIC value, which was the lowest at 43.76 (Supplementary Table S5). A Sankey diagram was drawn to show the relationship between five TCRLs and their related TCRGs (Figure 1F). The risk score was calculated as follows: risk score = (1.0662×AC092535.4) + (1.7218×AC087623.3) – (2.5683×PDCD4-AS1) + (1.5243×AL121820.2) + (1.9313×LINC01637).

**FIGURE 2:**
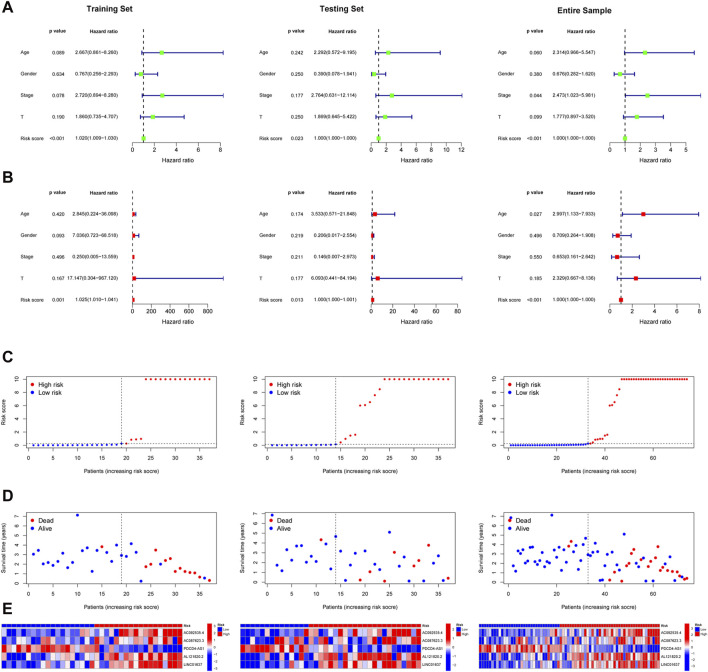
Validation of TCRL signature. The results of univariate Cox regression analysis **(A)** and multiple Cox regression analysis **(B)** of the training, testing, and entire sets; risk status **(C)**, survival outcome **(D)**, and expression levels of TCRLs in the signature **(E)**.

Patients in the training set, the testing sets, and entire samples were categorized into two subgroups (high-risk group and low-risk group) using the median risk score. Univariate Cox regression analysis showed that the risk score of UM patients was related to overall survival chance (Figure 2A). Multivariate Cox regression analysis demonstrated that the risk score could serve as an independent prognostic factor for UM patients (Figure 2B). The risk curves of all patients, including risk status, survival outcome, and expression of TCRLs in signature, are demonstrated in Figures 2C–E, respectively.

**FIGURE 3:**
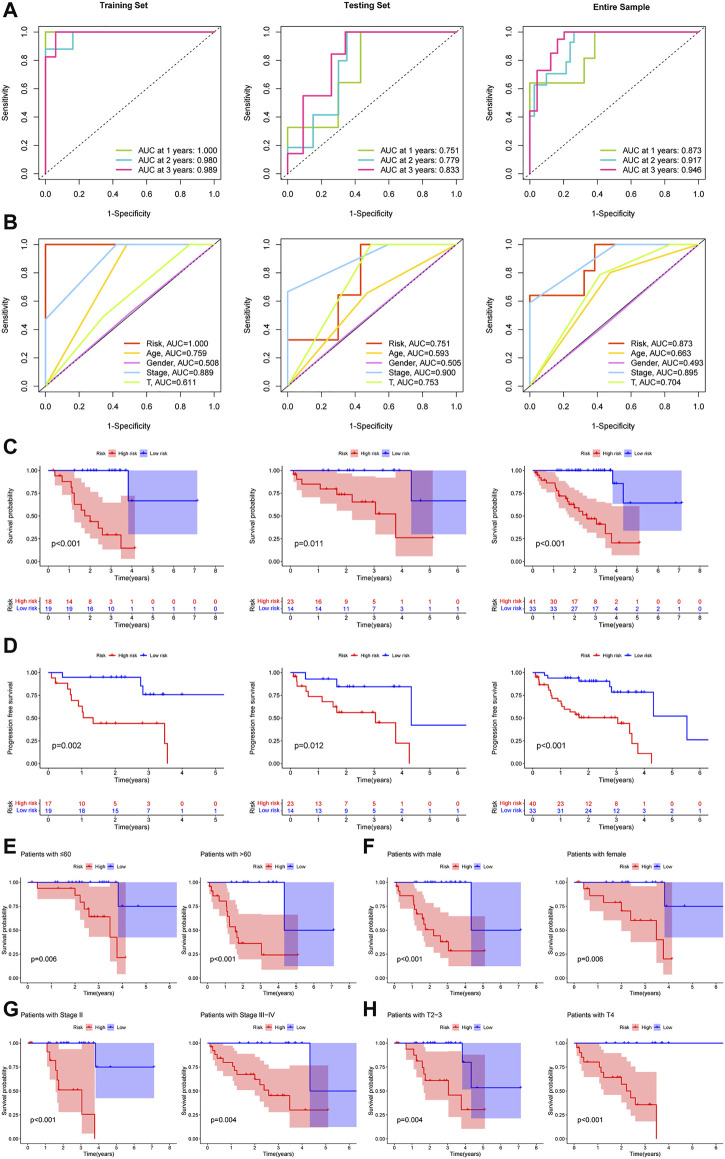
The prognostic capacity of the TCRL signature and the Kaplan–Meier survival curves of patients in subset groups. **(A)** The results of the 1-, 2-, and 3-year ROC curves of the training set, testing set, and entire sample. **(B)** ROC curves of risk scores and clinical characteristics. **(C)** Kaplan–Meier survival between the low- and high-risk groups. **(D)** Progression-free survival between the low- and high-risk groups. K-M survival curves of patients in age ≤60 years and >60 years **(E)**, in males and females **(F)**, in stage II and stage III–IV **(G)**, in T2-3 and T4 **(H)**.

Areas under the ROC curves of risk scores at 1, 2, and 3 years showed high prognostic prediction accuracy in three sets (Figure 3A). The ROC results also revealed that, compared to other clinical prediction models of UM, the TCRL signature can be a more robust prognostic indicator (Figure 3B). The K-M curve (Figure 3C) and progression-free survival (Figure 3D) showed that the high-risk group had the worst prognosis. Additionally, for equalizing the subset group size, we classified stage-II as early stage and stage-III and stage-IV as late stages. T2 and T3 were designated as locally progressive stages, while T4 was classified as an extra-scleral invasion stage (Ophthalmological Society of China Medicine Education AssociationOculoplastic and Orbital Disease Group of Chinese Ophthalmological Society of Chinese Medical AssociationOcular Oncology Committee of China Anti-Cancer Association, 2021). The K-M survival analysis was also conducted on subset groups separated by age, gender, stage, and T-stage (Figures 3E–H), and the results indicated that the overall survival of the high-risk group was shorter than that of the low-risk group. All results of the training set demonstrated that these five TCRLs are effective predictors of prognosis. This conclusion was further supported by the testing set and the entire sample.

To predict 1-, 2-, and 3-year survival rates of UM patients, we created a prognostic nomogram that combined clinicopathological parameters (gender, age, stage, and T-stage) with TCRL risk scores (Supplementary Figure S1A). Calibration curves were close to the 45 line, which demonstrated that the predictive nomogram had excellent calibration (Supplementary Figure S1B).

**FIGURE 4:**
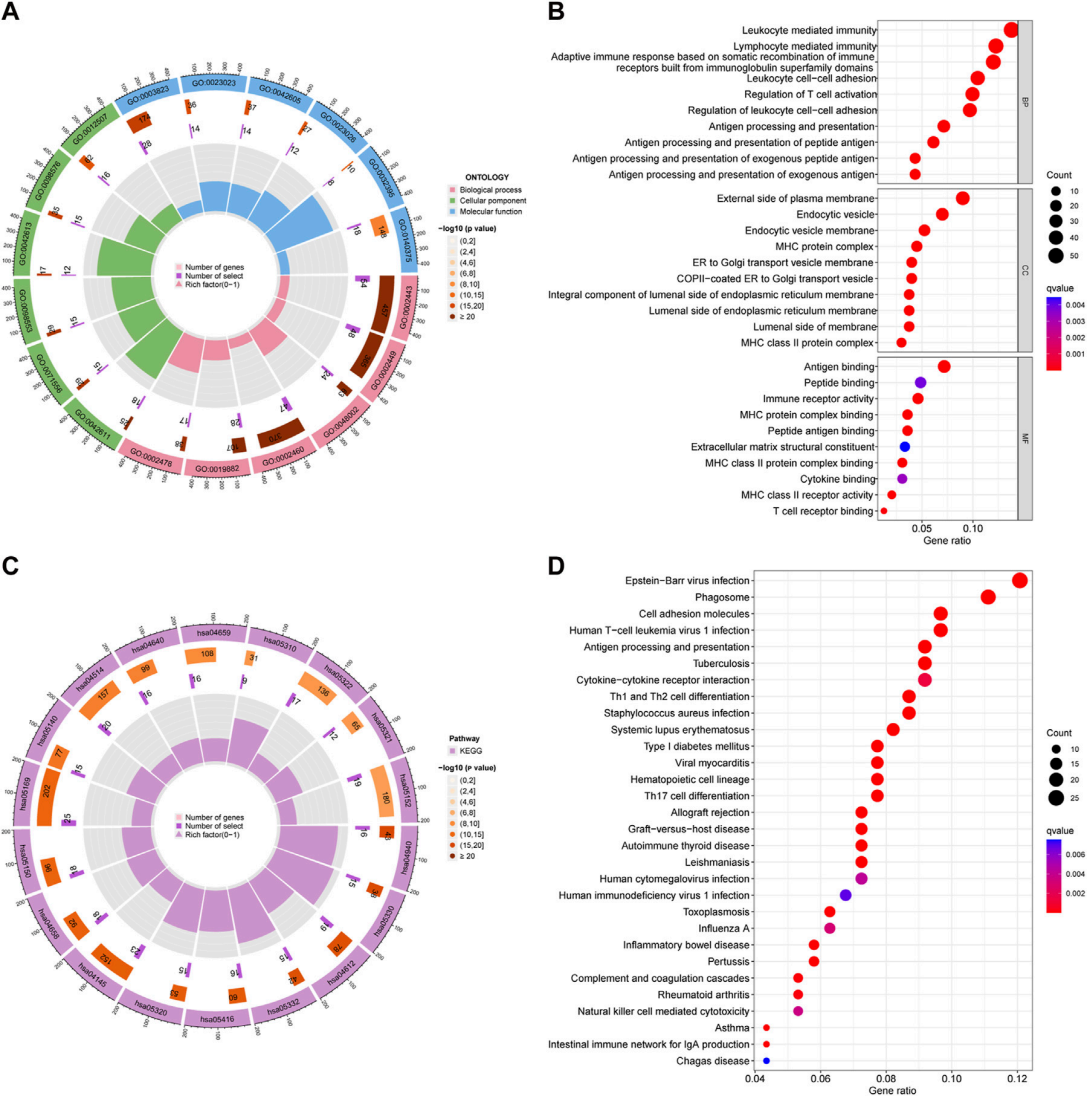
Results of GO and KEGG pathway enrichment analyses. **(A)** Circle diagrams of GO enriched pathways. **(B)** Bubble charts of GO enriched pathways. **(C)** Circle diagrams of KEGG enriched pathways. **(D)** Bubble charts of KEGG enriched pathways.

We employed differential expression analysis to identify 468 DEGs that differed in expression between the two groups (FDR < 0.05, |logFC| > 1.5). We conducted GO enrichment analysis to visualize the 10 primary molecular functions (MF), biological processes (BP), and cellular components (CC) using a circos diagram (Figure 4A) and a bubble diagram (Figure 4B). Specifically, the DEGs were enriched in leukocyte-mediated immunity, lymphocyte-mediated immunity, adaptive immune response based on somatic recombination of immune receptors built from immunoglobulin superfamily domains, and other functions of DEGs. The 30 primary KEGG pathways were represented by a circos diagram (Figure 4C) and a bubble diagram (Figure 4D). The DEGs enriched in Epstein−Barr virus infection, phagosome, and cell adhesion molecules were mainly activated.

Consensus clustering variables were created using the expression profiling of the five TCRLs, which was obtained from the entire sample. From the CDF curve and CDF delta area curve, we discovered that the most reliable clustering outcome was represented by k = 2 (Supplementary Figures S2A–C). To assess the reliability of the risk model, we conducted PCA (Supplementary Figure S2D) and t-SNE (Supplementary Figure S2E) analyses for all UM patients based on clustering results. There was clear distinction between the two clusters in every result. Then, we generated K-M survival curves of two clusters, which revealed that cluster one had better overall survival than cluster 2 (Supplementary Figure S2F).

**FIGURE 5:**
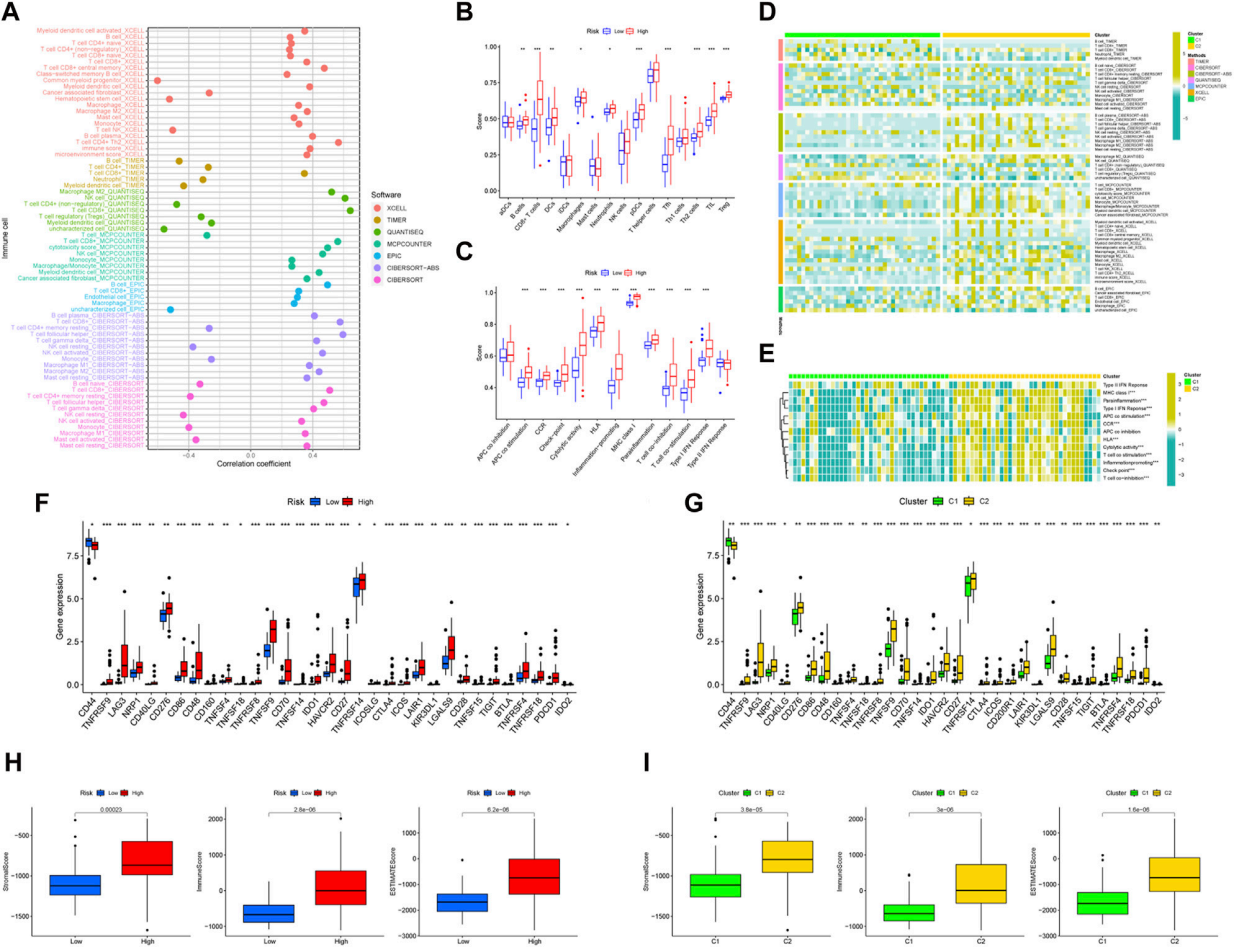
The immune landscape of UM. **(A)** Bubble diagram to verify the relationship between risk scores and immune components and heat map to demonstrate the distributions of infiltrating immune cell types in the two risk groups **(B)** and between two clusters **(C)**. Detailed differences in 13 immune-related functions were analyzed between the two risk groups **(D)** and two clusters **(E)**. Detailed differences in checkpoint genes were analyzed between the two risk groups **(F)** and two clusters **(G)**. The differences in stomal score, immune score, and ESTIMATE score between the two risk groups **(H)** and two clusters **(I)**.

We created a bubble diagram using the XCELL, TIMER, QUANTISEQ, MCPCOUNTER, EPIC, CIBERSORT-ABS, and CIBERSORT algorithms to demonstrate the relationship between risk scores and immune components (Figure 5A). Using the ssGSEA method, we plotted boxplots to reveal the differences in immune cell components and immune-related functions between the two risk groups (Figure 5B, C). We then plotted heatmaps to reveal the difference in immune cell components' immune-related functions between the two clusters (Figure 5D, E). We found that the high-risk group (Figure 5F) and the C2 cluster (Figure 5G) had more compared to immune cell infiltration and immune-related functions. Both risk groups and clusters were separately analyzed in terms of differences in immune checkpoint genes. All immune checkpoint genes appeared to be significantly different, the majority of which were highly expressed in the high-risk group compared to the low-risk group. The risk groups (Figure 5H) and clusters (Figure 5I) were separately evaluated using boxplots in terms of differences in the content of immune and stromal cells. The results showed that the UM patients in the high-risk group and cluster 2 had higher than immune, stromal, and ESTIMATE scores.

The high-risk group presented significantly lower TIDE scores than the low-risk group (Supplementary Figure S3A), which means that the high-risk group may have a better immunotherapy effect. By using the pRRophetic package (Geeleher et al., 2014), 28 anticancer drugs whose sensitivity (IC50) was significantly related to the model were screened out. It is worth noting that several kinds of tumor chemotherapy drugs and targeted drugs have higher sensitivity in the high-risk group (for example, gefitinib, mitomycin C, and temsirolimus) (Supplementary Figure S3B). Among the 28 anticancer drugs, 9 showed higher sensitivity in the high-risk group, while 19 had lower sensitivity in the high-risk group.

**FIGURE 6:**
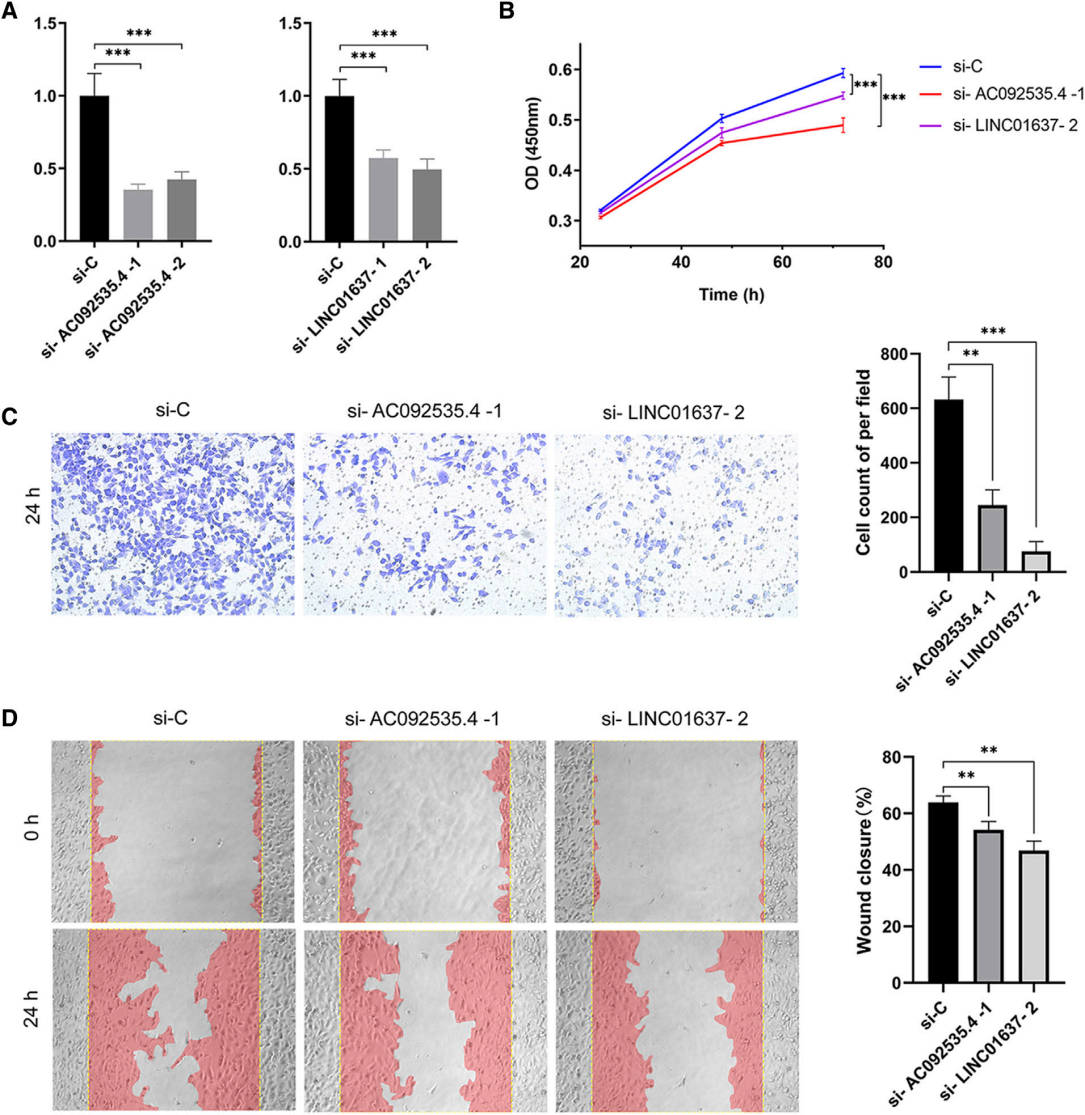
Gene function verification experiment. **(A)** Verification of gene silencing using qRT-PCR. **(B)** Proliferation by CCK8 assay. **(C)** Representative images (×10) and cell counting results of transwell invasion assay. **(D)** Representative images (×10) and the wound closure rate of scratch wound assay. Data are shown as mean (standard deviation [SD]); ***p ≤ 0.001 or **p ≤ 0.01 compared to the control.

In order to minimize the impact of the limited sample size on the reliability of the results, two approaches were employed. Firstly, the OSuvm database, an online consensus survival tool for prognostic analysis of uveal melanoma, was searched. Despite the limited number of TCRLs (PDCD4-AS1 and LINC01637) retrieved (Wang et al., 2020), K-M survival analysis of these two TCRLs demonstrated statistically significant differences (Supplementary Figure S4), thereby confirming that PDCD4-AS1 is a beneficial gene while LINC01637 is a risk gene. Secondly, the machine learning technique of Random Forest analysis was employed to obtain 14 TCRLs based on univariate Cox analysis (Supplementary Table S6). It is noteworthy that all five TCRLs previously obtained through Lasso-Cox analysis were included in this approach. Consequently, the five TCRLs identified in the Lasso-Cox model were deemed reliable candidate biomarkers for UM prognosis. In order to identify more reliable prognostic biomarkers, the model was validated using univariate and multivariate Cox analyses, which resulted in the identification of 10 TCRLs (Supplementary Table S7). The intersection of these with the five TCRLs from the Lasso-Cox model yielded two TCRLs (AC092535.4 and LINC01637). To further explore the potential cell function of AC092535.4 and LINC01637, the MUM2B cell line was constructed using lncRNA knockdown phenotypes. The transfection efficiency was confirmed using qRT-PCR (Figure 6A), and both siRNA fragments dramatically reduced the expression of AC092535.4 and LINC01637. Next, we performed a series of function assays. The CCK-8 assay demonstrated that reduced expression of either AC092535.4 or LINC01637 inhibited the proliferative capacity of MUM2B (Figure 6B). In addition, the under-expression of AC092535.4 and LINC01637 reduced the invasion ability of MUM2B cells in the transwell invasion assay (Figure 6C) and decreased their migratory ability in the scratch experiment (Figure 6D). These findings imply that AC092535.4 and LINC01637 serve as high-risk predictors in MUM2B cells, which have complex effects on the growth of UM.

## 4 Discussion

It is a well-known fact that TRP channels act as a crucial part of the calcium signaling machinery. TRP channels, which have been observed in many immune system cells (Bertin et al., 2014), are involved in the activation and maintenance of immune systems. GO enrichment analysis pointed to leukocyte-mediated immunity, lymphocyte-mediated immunity, regulation of T-cell activation, and T-cell receptor binding. GO and KEGG enrichment analyses included antigen processing and presentation. Previous studies have indicated that TRPV2 participated in the chemotaxis and phagocytosis of macrophages (Link et al., 2010), TRPM2 participated in the activation of macrophages and T-cells (Feske et al., 2015), and TRPM and TRPV participated in the antigen presentation of dendritic cells (Froghi et al., 2021). These results were consistent with our enrichment analysis results. It also illustrated that there was a broad research space for TRP channels regulated UM immune cell functions.

Tumor-infiltrating immune cells were crucial for the treatment responsiveness and prognosis of cancer (Fridman et al., 2012). Previous research has suggested that there are multiple tumor-infiltrating immune cells in UM, such as CD8^+^ T-cells, CD4^+^ Treg, and macrophages (Maat et al., 2009). In this study, ssGSEA was employed to elucidate the correlation between five TCRLs expression and immune infiltration. The findings revealed that the composition of immune cells, immune-related functions, and immune checkpoints are significantly elevated in the high-risk group in comparison to the low-risk group. Furthermore, a stronger inflammatory response and immune cell activity were observed in the high-risk population through the characterization of the tumor microenvironment. The aforementioned assessment of the tumor immune microenvironment has revealed that tumor cells in the UM high-risk population are activated by the immune system. This activation may have a positive impact on immunotherapy and potentially beneficial in counteracting tumor immune escape. Previous studies have demonstrated a positive correlation between AC092535.4 and the number of regulatory T cells (Zhang et al., 2021a). Our study revealed that individuals with high-risk scores for TCRLs exhibited higher regulatory T cell levels. However, further investigation is required to ascertain whether a correlation exists. Because the uvea is immunologically privileged, UM is distinct from cancer cells from other tissue sources (Forrester and Xu, 2012). Despite its microenvironment being infiltrated with immune cells, UM has been believed to have avoided immune surveillance (Niederkorn, 2012). Limited immune surveillance has exposed UM to a dormant state regulated by CD8^+^ T-cells for a significant time (Eyles et al., 2010). Patients of UM relapsed and metastasized after 5 years (Li et al., 2020), which increased the difficulty of the immunotherapy. Nevertheless, based on the active T cell repertoires present in UM patients (Boudousquie et al., 2017), a study revealed that direct contact between induced T cells and tumor cells can form an immunological synapse, which causes cancer cell lysis (Majhi et al., 2015). This has opened new avenues for the immunotherapy of UM.

Since immune checkpoint inhibitors modulate antitumor immune responses, they are widely used in the treatment of tumors (Chae et al., 2018). There is no denying that immunotherapy has greatly improved the prognosis of patients with cutaneous melanoma (Riley et al., 2019). However, due to the differences in treatment modalities between UM and cutaneous melanoma, the use of immune checkpoint inhibitors in the treatment of primary UM remains controversial (Carvajal et al., 2017). For this reason, we analyzed the immune checkpoints and demonstrated that there were significant differences in the 33 immune checkpoints between the compared groups. We specifically focused on PDCD1 and CTLA4, both of which were highly expressed in the high-risk group and cluster 2. Masaoutis et al. (2021) suggested that using PDCD1-and CTLA-4-blocking antibodies appeared to be the current best treatment option for metastatic UM. Additionally, we observed that the TIDE score of the high-risk group was lower than that of the low-risk group, revealing that high-risk patients may benefit more from immune checkpoint inhibitor therapy.

Screening for effective chemotherapeutic drugs based on the sensitivity of drugs is the current research hotspot in personalized cancer treatment. Previous research has found that ipilimumab is beneficial to advanced UM patients (Maio et al., 2013). A higher expression of cytochrome P450 reductase in UM makes UM more sensitive to mitomycin C (Gravells et al., 2011). TRP channels can act as both targeted ion channels and drug carriers, as they are expressed by many cancer cells. Existing research studies have suggested that the TRPV6 channel is regulated by androgens; therefore, bicalutamide, an androgen antagonist, has been widely used in the treatment of advanced prostate cancer. When used as drug carriers, TRP channels selectively introduce cytotoxic charged molecules into cancer cells. Doxorubicin has been selected as an excellent candidate drug based on its low molecular weight and positive charge. Our study found that high-risk patients are more sensitive to gefitinib, mitomycin C, and temsirolimus compared to low-risk patients. This may provide valuable novel insights into TRP channel-based therapy for UM patients.

All five of the candidate lncRNAs (AC092535.4, AC087623.3, PDCD4-AS1, AL121820.2, and LINC01637) were discovered to be related with UM for the first time. It is worth noting that AC092535.4 and PDCD4-AS1 have been proposed as biomarkers for certain cancer types. Zhang et al. (2021b) found that clear cell renal cell carcinoma patients with high AC092535.4 expression presented significantly low survival rates, illustrating that AC092535.4 promotes tumor growth. Additionally, PDCD4—a novel tumor suppressor gene—had a low level of expression in multiple cancer types, such as lung, colorectal, hepatocellular, and breast cancers (Chen et al., 2003; Mudduluru et al., 2007). PDCD4-AS1 has been shown to act upstream of PDCD4 to regulate its expression. The overexpression of PDCD4-AS1 can inhibit cancer cell proliferation and increase the cancer cell apoptosis rate (Zhang et al., 2006). Because few studies have reported AC087623.3, AL121820.2, and LINC01637, we did not have access to relevant information. In this study, CCK8 results showed that downregulated expression of AC092535.4 and LINC01637 reduced the proliferation of UM cells, while downregulated expression of PDCD4-AS1 promoted the proliferation of UM cells. Similarly, knocking out AC092535.4 and LINC01637 can inhibit the migration and invasion of UM cells and delay the healing rate. The findings suggest that AC092535.4 and LINC01637 may serve as novel prognostic biomarkers for UM. The specific mechanisms of intervention require further study.

However, this article has certain limitations. First, the accuracy of our results and prediction can be improved if there are more samples and more complete clinical information. Second, we did not collect clinical data to validate the nomogram.

## 5 Conclusion

The expression of AC092535.4 and LINC01637 was found to be significantly associated with poor prognosis and immune infiltration in UM. These findings indicate that AC092535.4 and LINC01637 may be valuable prognostic biomarkers for UM. These findings provide insights that can be utilized to further elucidate the potential functions of AC092535.4 and LINC01637 in UM. Additionally, they may serve as promising individualized therapeutic targets for UM treatment.

## Data Availability

The datasets presented in this study can be found in online repositories. The names of the repository/repositories and accession number(s) can be found in the article/Supplementary Material.
