# Supplementary material for: TRP channel-related LncRNAs, AC092535.4 and LINC01637, as novel prognostic biomarkers for uveal melanoma
Source: Front Genet. 2024 Jul 23;15:1441732. doi: 10.3389/fgene.2024.1441732 (PMC11300240; doi:10.3389/fgene.2024.1441732)
Supplement: Supplementary file 1 [file Table1.docx]

Supplementary Materials

**Supplementary Table 1.** The following primers were used in qRT-PCR.

**Supplementary Table 2.** siRNA sequences.

**Supplementary Table 3.** TCRGs.

**Supplementary Table 4.** Characteristics of 74 uveal melanoma patients.

**Supplementary Table 5.** Akaike information criterion for the prognostic signature.

**Supplementary Table 6.** Univariate Cox analysis and Random Forest analysis.

**Supplementary Table 7.** Univariate and multivariate Cox analyses to verify the model.

**Supplementary Figure 1.** Development of a prognostic nomogram.

**Supplementary Figure 2.** Identification of five TCRL-related clusters.

**Supplementary Figure 3.** Immunotherapy and drug sensitivity analysis.

**Supplementary Figure 4.** The K-M survival analysis of the two TCRLs in OSuvm database.

# Supplementary Tables

## Supplementary Table 1

| **Supplementary Table 1 The following primers were used in qRT-PCR:** | | |
| --- | --- | --- |
| AC092535.4 | forward (5'-3') | ATCGCCCTGGTCCAGACA |
|  | reverse (5'-3') | GTCAGCGTAGTGGCTCCCTT |
| LINC01637 | forward (5'-3') | CTGAAAACGGGCTCGGAC |
|  | reverse (5'-3') | AAGGTCTGGAGGACACGGAG |

**Supplementary Table 1.** The following primers were used in qRT-PCR.

## Supplementary Table 2

| **Supplementary Table 2 siRNA sequences are as follows:** | |
| --- | --- |
| si- AC092535.4- 1 | GTCCTCAGAATGAAATCCA |
| si- AC092535.4- 2 | CCACTACGCTGACTTCTAA |
| si- LINC01637- 1 | GTAAATGACAGGTGAGAAA |
| si- LINC01637- 2 | CTACCTACAACGCAGAGAT |

**Supplementary Table 2.** siRNA sequences.

## Supplementary Table 3

| **Supplementary Table 3 TCRGs** |
| --- |
| MCOLN3 |
| ADCY1 |
| ADCY2 |
| ADCY3 |
| ADCY4 |
| ADCY5 |
| ADCY6 |
| ADCY7 |
| ADCY8 |
| ADCY9 |
| ALOX12 |
| ASIC1 |
| ASIC2 |
| ASIC3 |
| ASIC4 |
| ASIC5 |
| BDKRB1 |
| BDKRB2 |
| CALM1 |
| CALM2 |
| CALM3 |
| CALML3 |
| CALML4 |
| CALML5 |
| CALML6 |
| CAMK2A |
| CAMK2B |
| CAMK2D |
| CAMK2G |
| CYP2J2 |
| F2RL1 |
| GNAQ |
| GNAS |
| HRH1 |
| HTR2A |
| HTR2B |
| HTR2C |
| IGF1 |
| IL1B |
| IL1R1 |
| IL1RAP |
| ITPR1 |
| ITPR2 |
| ITPR3 |
| JMJD7-PLA2G4B |
| KNG1 |
| MAP2K3 |
| MAP2K6 |
| MAPK10 |
| MAPK11 |
| MAPK12 |
| MAPK13 |
| MAPK14 |
| MAPK8 |
| MAPK9 |
| MCOLN1 |
| MCOLN2 |
| MLKL |
| NGF |
| NTRK1 |
| P2RY2 |
| PIK3CA |
| PIK3CB |
| PIK3CD |
| PIK3R1 |
| PIK3R2 |
| PIK3R3 |
| PLA2G4A |
| PLA2G4B |
| PLA2G4C |
| PLA2G4D |
| PLA2G4E |
| PLA2G4F |
| PLA2G6 |
| PLCB1 |
| PLCB2 |
| PLCB3 |
| PLCB4 |
| PLCG1 |
| PLCG2 |
| PPP1CA |
| PPP1CB |
| PPP1CC |
| PRKACA |
| PRKACB |
| PRKACG |
| PRKCA |
| PRKCB |
| PRKCD |
| PRKCE |
| PRKCG |
| PRKCH |
| PRKCQ |
| PTGER2 |
| PTGER4 |
| RIPK1 |
| RIPK3 |
| SRC |
| TRPA1 |
| TRPC1 |
| TRPC3 |
| TRPC4 |
| TRPC4AP |
| TRPC5 |
| TRPC6 |
| TRPC7 |
| TRPM1 |
| TRPM2 |
| TRPM3 |
| TRPM4 |
| TRPM5 |
| TRPM6 |
| TRPM7 |
| TRPM8 |
| TRPV1 |
| TRPV2 |
| TRPV3 |
| TRPV4 |
| TRPV5 |
| TRPV6 |

**Supplementary Table 3.** TCRGs.

## Supplementary Table 4

| **Supplementary Table 4  Characteristics of 74 uveal melanoma patients** | | | | | |
| --- | --- | --- | --- | --- | --- |
| **Characteristics** | **Type** | **Entire set** | **Training set** | **Testing set** | ***p*-value** |
| Age | ＜=60 | 37 (50%) | 18 (48.65%) | 19 (51.35%) | 0.802 |
|  | >60 | 37 (50%) | 19 (51.35%) | 18 (48.65%) |  |
| Gender | Male | 42 (56.76%) | 18 (48.65%) | 24 (64.86%) | 0.159 |
|  | Female | 32 (43.24%) | 19 (51.35%) | 13 (35.14%) |  |
| Stage | II | 35 (47.30%) | 21 (56.76%) | 14 (37.84%) | 0.247 |
|  | III | 35 (47.30%) | 14 (37.84%) | 21 (56.76%) |  |
|  | IV | 4 (5.41%) | 2 (5.41%) | 2 (5.41%) |  |
| T | 2a/2b | 12 (16.22%) | 5 (13.51%) | 7 (18.92%) | 0.100 |
|  | 3a/3b/3c | 29 (39.19%) | 19 (51.35%) | 10 (27.03%) |  |
|  | 4a/4b/4c/4d/4e | 33 (44.59%) | 13 (35.14%) | 20 (54.05%) |  |

**Supplementary Table 4.** Characteristics of 74 uveal melanoma patients.

## Supplementary Table 5

| **Supplementary Table 5**  **Akaike information criterion for the prognostic signature** | | |
| --- | --- | --- |
| **Model** | **Prognostic signature combination** | **AIC** |
| 1 | AC092535.4 + AC087623.3 + PDCD4-AS1 + AL121820.2 + AC015818.2 + SNHG16 + ZNF667-AS1 + LINC01637 | 48.8 |
| 2 | AC092535.4 + AC087623.3 + PDCD4-AS1 + AL121820.2 + AC015818.2 + SNHG16 + LINC01637 | 46.8 |
| 3 | AC092535.4 + AC087623.3 + PDCD4-AS1 + AL121820.2 + AC015818.2 + LINC01637 | 44.83 |
| 4 | AC092535.4 + AC087623.3 + PDCD4-AS1 + AL121820.2 + LINC01637 | 43.76 |

**Supplementary Table 5.** Akaike information criterion for the prognostic signature.

## Supplementary Table 6

| **Supplementary Table 6**  **Univariate Cox analysis and Random Forest analysis** | |
| --- | --- |
| **LncRNA** | **Score** |
| **AC092535.4** | 0.066319 |
| LINC00963 | 0.050155 |
| AC103974.1 | 0.03973 |
| **LINC01637** | 0.0194 |
| AC017104.1 | 0.034052 |
| **PDCD4-AS1** | 0.016277 |
| AC016747.1 | 0.024185 |
| AC103706.1 | 0.020444 |
| AC008556.1 | 0.010057 |
| **AL121820.2** | 0.010988 |
| **AC087623.3** | 0.005888 |
| AC018904.1 | 0.027989 |
| AC008736.1 | 0.01925 |
| MIR22HG | -0.00285 |

**Supplementary Table 6.** Univariate Cox analysis and Random Forest analysis.

## Supplementary Table 7

| **Supplementary Table 7**  **Univariate and multivariate Cox analyses to verify the model** | |
| --- | --- |
| **LncRNA** | ***p* value** |
| **AC097359.2** | 3.21E-108 |
| AC092535.4 | 8.71E+61 |
| AL157702.2 | 2.56E+250 |
| LINC00963 | 7.00E+213 |
| SNHG7 | 1.21E+91 |
| AC136475.3 | 1.06E-158 |
| AC015818.2 | 7.67E+130 |
| AC018529.1 | 2.61E-25 |
| AC008556.1 | 3.61E-159 |
| **LINC01637** | 7.35E+153 |

**Supplementary Table 7.** Univariate and multivariate Cox analyses to verify the model.

# Supplementary Figures

## Supplementary Figure 1


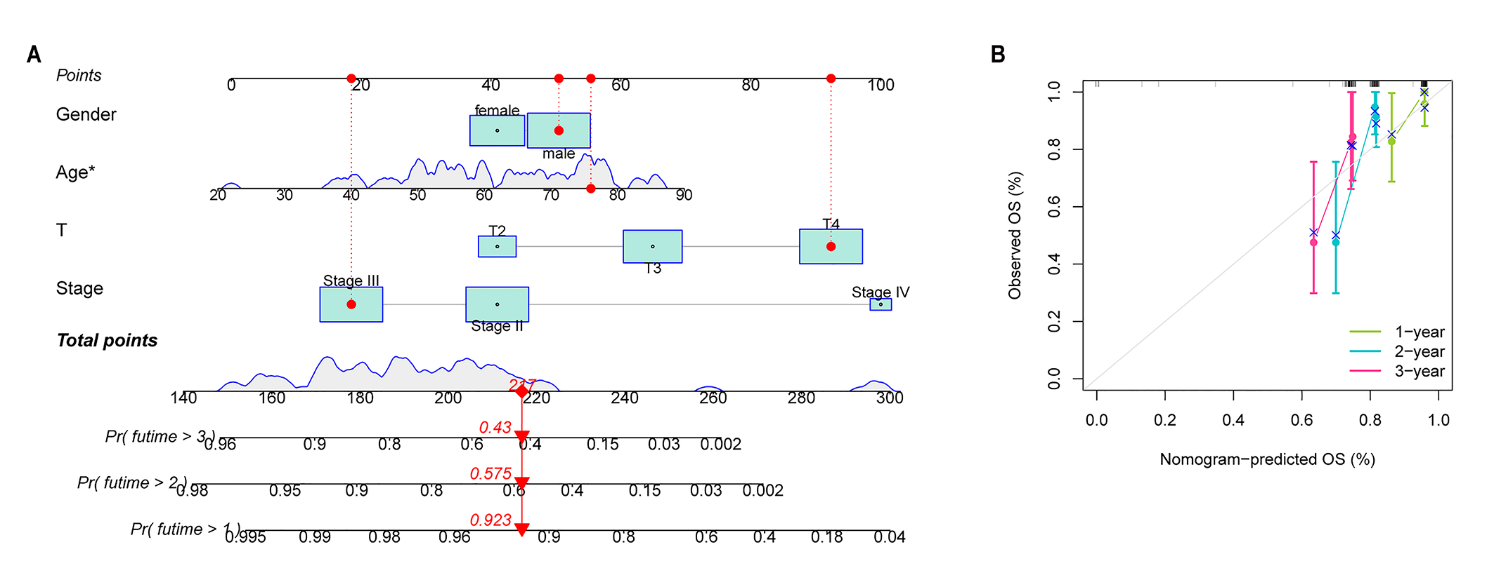


**Supplementary Figure 1.** Development of a prognostic nomogram. **(A)** Nomogram of the prognostic model based on TCRL signature and clinicopathological characteristics. **(B)** Calibration curve analysis of the 1-, 2-, and 3-year survival prediction accuracy of the nomogram.

## Supplementary Figure 2

**
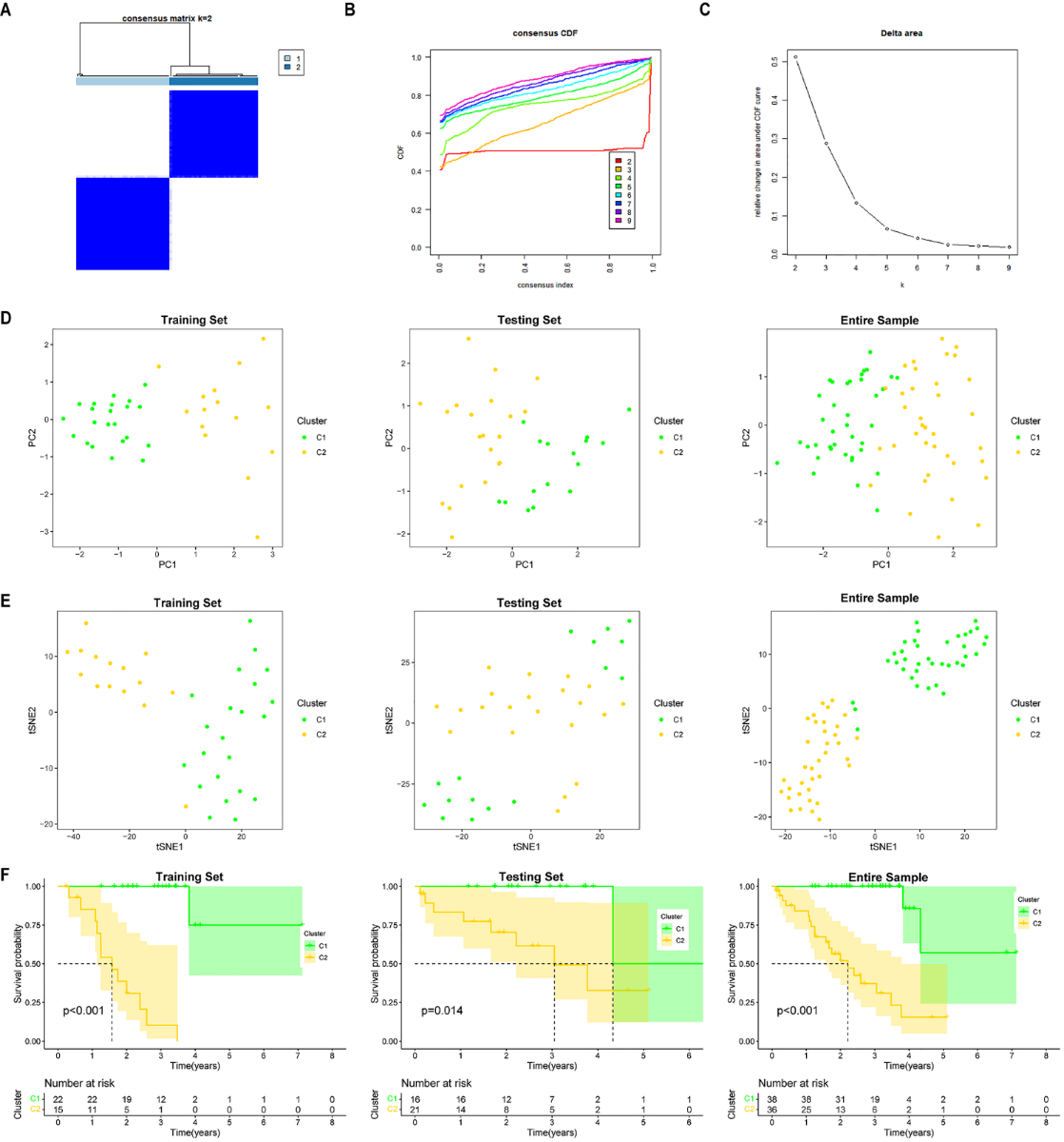
**

**Supplementary Figure 2.** Identification of five TCRL-related clusters. **(A)** The consensus score matrix (k = 2) of 74 UM samples by consensus cluster analysis. **(B)** CDF curves for cluster number k = 2-9. **(C)** Relative change in the area under the CDF curve for k = 2-9. The plot of PCA results **(D)**, the t-SNE analysis **(E)**, and the K-M plot **(F)** for two clusters of the training set, testing set, and the entire sample.

## Supplementary Figure 3

**
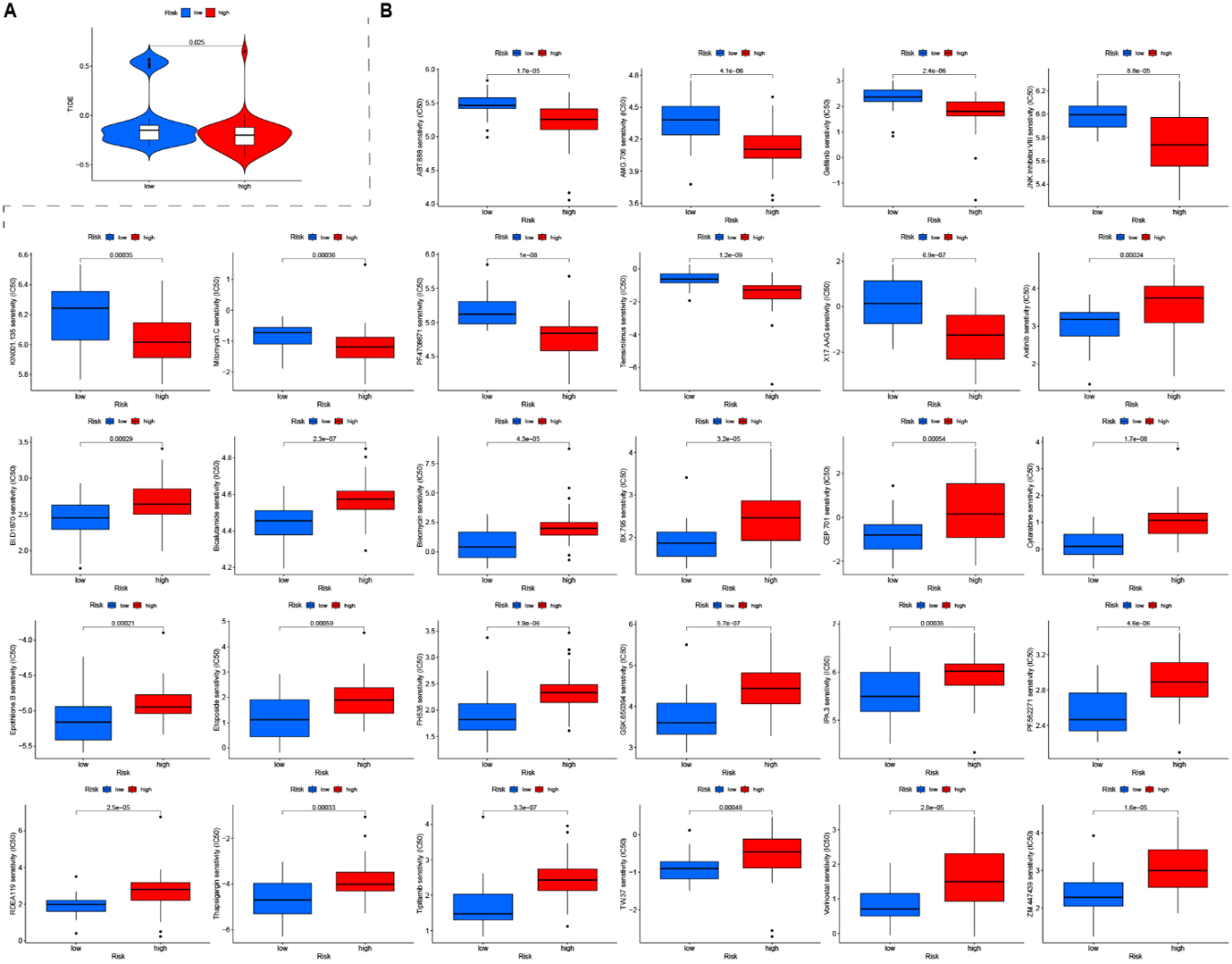
**

**Supplementary Figure 3.** Immunotherapy and drug sensitivity analysis. Violin plot to illuminate the difference in TIDE between the two risk groups **(A)**. Comparison of IC50 of anti-tumor drugs between risk groups **(B)**.

## Supplementary Figure 4


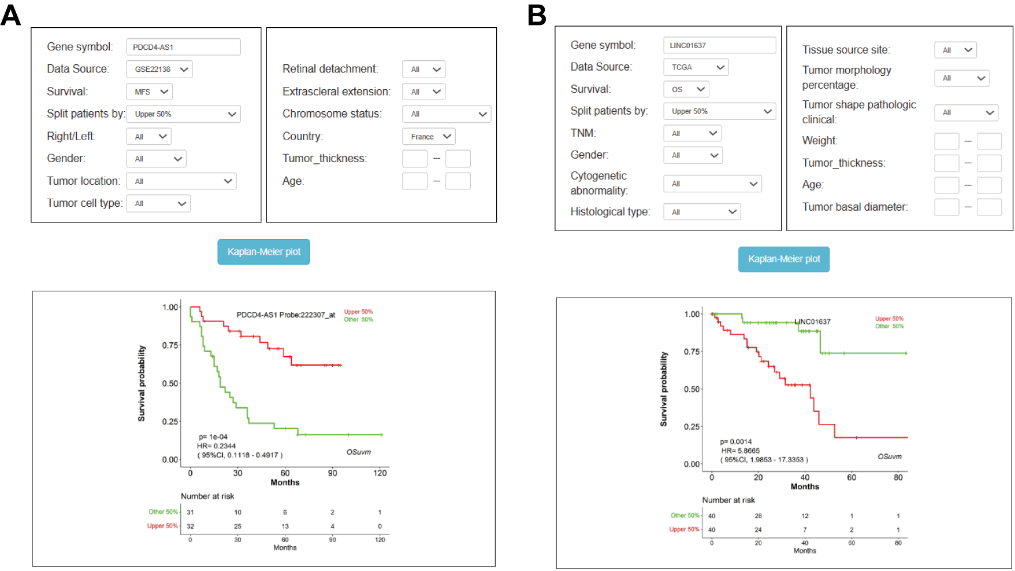


**Supplementary Figure 4.** The K-M survival analysis of the two TCRLs in OSuvm database. **(A)** The K-M survival analysis of PDCD4-AS1; **(B)** The K-M survival analysis of LINC01637.
